# Untargeted ^1^H-NMR Metabolomics Identifies Candidate Metabolic Changes Associated with Watercress (*Nasturtium officinale* R.Br.) Supplementation in Adults with Low-to-Moderate Cardiovascular Risk: A Randomized Placebo-Controlled Trial

**DOI:** 10.3390/nu18152559

**Published:** 2026-08-05

**Authors:** Chikondi Maluwa, Blecious Zinan’dala, Praporn Kijkuokool, Puriwat Fakfum, Churdsak Jaikang, Giatgong Konguthaithip, Wason Parklak, Hataichanok Chuljerm, Kanokwan Kulprachakarn

**Affiliations:** 1 School of Health Sciences Research, Research Institute for Health Sciences, Chiang Mai University, Chiang Mai 50200, Thailand; chikondi_maluwa@cmu.ac.th (C.M.); dysonblecious_zina@cmu.ac.th (B.Z.); praporn_k@cmu.ac.th (P.K.); puriwat_f@cmu.ac.th (P.F.); wason.p@cmu.ac.th (W.P.); hataichanok.ch@cmu.ac.th (H.C.); 2 Faculty of Clinical Sciences, Malawi College of Health Sciences, Lilongwe P.O. Box 30368, Malawi; 3 Metabolomic Research Group for Forensic Medicine and Toxicology, Department of Forensic Medicine, Faculty of Medicine, Chiang Mai University, Chiang Mai 50200, Thailand; churdsak.j@cmu.ac.th (C.J.); kongkiat.shang@gmail.com (G.K.)

**Keywords:** watercress, *Nasturtium officinale*, metabolomics, ^1^H-NMR spectroscopy, cardiovascular risk, glucosinolates, metabolic pathway

## Abstract

**Background/Objectives:** Watercress (*Nasturtium officinale* R.Br.) is a glucosinolate-rich cruciferous vegetable with reported cardioprotective properties. However, previous human studies have relied on targeted clinical and biochemical biomarkers, limiting insight into its broader metabolic effects. This exploratory study investigated plasma metabolomic changes associated with watercress supplementation in adults with low-to-moderate cardiovascular risk using untargeted proton nuclear magnetic resonance (^1^H-NMR) spectroscopy. **Methods:** In this randomized, single-blind, placebo-controlled pilot trial (Thai Clinical Trials Registry: TCTR20251119004), 26 participants aged 40–59 years received dried watercress capsules (8 g/day; approximately 195 mg glucosinolates/day) or placebo for 28 days. Fasting plasma samples collected at baseline and Day 28 underwent untargeted ^1^H-NMR profiling. Partial least squares-discriminant analysis (PLS-DA) assessed group discrimination, while Kyoto Encyclopedia of Genes and Genomes (KEGG)-based pathway enrichment and topology analyses identified perturbed metabolic pathways. **Results:** A total of 209 plasma metabolites were identified. PLS-DA demonstrated modest discrimination between groups (Q^2^ = 0.268), with 27 discriminant metabolites (variable importance in projection > 1.5) involving amino acid, nucleotide, carbohydrate, and gut microbial metabolism. Eighteen metabolic pathways were significantly perturbed, particularly galactose and fructose/mannose metabolism (*p* < 0.001), together with glycerolipid, purine, glycolysis/gluconeogenesis, and tryptophan metabolism. Watercress supplementation reduced metabolites associated with oxidative DNA damage, inflammatory kynurenine metabolism, and microbial co-metabolism, while increasing glycine and dimethylglycine and altered gut microbial activity. Conventional biomarkers showed a significant decrease in low-density lipoprotein cholesterol but no consistent effects on other lipids. **Conclusions:** Watercress supplementation was associated with coordinated metabolic alterations across multiple pathways, generating mechanistic hypotheses for its antioxidant and cardiometabolic effects. These findings are exploratory and warrant confirmation in larger, adequately powered clinical trials.

## 1. Introduction

Cardiovascular disease (CVD) remains the leading cause of death globally, accounting for approximately 17.9 million deaths annually, with the greatest burden occurring in low- and middle-income countries [1]. This trend is mirrored in Thailand, where CVD-attributable mortality is estimated at 23% per year [2,3], and the prevalence of metabolic syndrome is estimated at 24–32% among Thai adults [4]. The principal modifiable risk factors for CVD, hypertension, dyslipidemia, hyperglycemia, and obesity are themselves driven by unhealthy dietary patterns, physical inactivity, tobacco use, and excess alcohol consumption [5]. Given that individuals at low-to-moderate cardiovascular risk represent the largest reservoir from which future CVD events arise [6], early, low-cost, and scalable dietary interventions in this population remain a public-health priority, particularly given that pharmacotherapy is typically reserved for those at intermediate or high risk [7].

Dietary modification, particularly increased consumption of vegetables, fruit, whole grains, and antioxidant-rich foods, is consistently recommended as a first-line, non-pharmacological strategy for cardiovascular risk reduction [8]. Among commonly consumed vegetables, watercress (*Nasturtium officinale* R.Br.) has attracted considerable attention owing to its exceptional nutritional value. It is one of the foods with the highest nutrient density score, surpassing other cruciferous and leafy green vegetables, including kale, chard, and spinach [9]. Watercress is a semi-aquatic, perennial member of the Brassicaceae family distributed across Europe, Asia, and Africa, and is also cultivated domestically in Thailand [10]. Beyond its conventional nutrient content, watercress is a particularly rich source of glucosinolates (notably gluconasturtiin, the precursor of the bioactive isothiocyanate phenethyl isothiocyanate, PEITC), dietary nitrate, ascorbic acid, carotenoids, vitamins and phenolic compounds, which together support much of its proposed cardioprotective and chemopreventive bioactivity [11].

Mechanistic and human intervention data have shown several plausible cardiometabolic benefits of watercress. PEITC and related isothiocyanates activate the nuclear factor erythroid 2-related factor 2 (Nrf2) antioxidant-response pathway, inducing cytoprotective enzymes that limit low-density lipoprotein oxidation and endothelial oxidative stress [12], while watercress’s substantial dietary nitrate content can be sequentially reduced to nitrite and nitric oxide via the nitrate–nitrite–nitric oxide pathway, promoting vasodilation and blood-pressure reduction; an inhibitory action on angiotensin-converting enzyme has also been reported for watercress extracts [13,14]. Further evidence suggests that watercress supports the detoxification of environmental toxicants and carcinogens through the activation of phase II detoxification enzymes resulting in the production of glutathione S-transferases (GSTs) [15]. In an earlier randomized, placebo-controlled crossover trial, eight weeks of daily watercress supplementation reduced lymphocyte DNA damage and altered plasma antioxidant status in healthy adults [16]. More recently, a randomized, single-blinded, placebo-controlled pilot trial in middle-aged Thai adults with low-to-moderate cardiovascular risk, the parent clinical trial from which the present cohort and biospecimens were drawn, found that four weeks of daily dried-watercress-capsule consumption (providing 195 mg/day of glucosinolate) significantly lowered systolic and diastolic blood pressure relative to placebo, alongside within-group improvements in oxidized LDL and total antioxidant capacity that did not reach statistical significance between groups, and a significant within-group reduction in estimated 10-year Framingham cardiovascular risk [17].

These previous studies relied on targeted, hypothesis-driven clinical and biochemical biomarkers, including blood pressure, lipid profile, oxidized LDL, and antioxidant capacity. Although informative, such targeted approaches are inherently limited to predefined analytes and therefore cannot capture the broader metabolic perturbations and interconnected biochemical networks. Untargeted metabolomics offers a complementary, hypothesis-generating approach to this problem, by simultaneously profiling hundreds of low-molecular-weight metabolites spanning amino acid, nucleotide, lipid, and carbohydrate metabolism. It can reveal coordinated, unanticipated shifts that would not be captured by routine biochemistry and can generate mechanistic hypotheses for subsequent targeted confirmation [18,19]. Proton nuclear magnetic resonance (^1^H-NMR) spectroscopy is a robust, highly reproducible, and minimally destructive platform well suited to this purpose in plasma, requiring little sample preparation although its metabolite coverage and analytical sensitivity are lower than those achievable with liquid chromatography–mass spectrometry (LC-MS). Nevertheless, its robustness and quantitative reproducibility make it an attractive platform for exploratory nutritional intervention studies of modest size [20].

The present investigation represents a pre-planned exploratory metabolomics analysis of archived plasma samples collected during our previously reported randomized, single-blind, placebo-controlled pilot trial [17]. Although the parent study was designed primarily to evaluate cardiovascular outcomes, paired plasma samples obtained before and after the intervention provided an opportunity to explore metabolic alterations associated with watercress supplementation. Hence, the objectives of this exploratory analysis were to characterize changes in the circulating plasma metabolome using untargeted ^1^H-NMR metabolomics and to identify candidate metabolites and metabolic pathways associated with watercress supplementation. Given the pilot nature of the study, the modest sample size, and the semi-quantitative characteristics of untargeted ^1^H-NMR metabolomics, the findings are intended to generate biological hypotheses and guide future targeted metabolomic and mechanistic investigations rather than establish causal mechanisms.

## 2. Materials and Methods

### 2.1. The Intervention

A single batch of fresh watercress was procured from Zhi Wu Watercress Farm, Yala Province, Thailand, in December 2024 to ensure batch consistency throughout the intervention. The watercress was dried by roasting at 50 °C, finely ground, and encapsulated into size 0 hard gelatin capsules containing 500 mg of powder each. Participants in the intervention group received an oral dose of 8 g/day. Based on previous compositional analysis of watercress processed using the same roasting method, the dried powder contained 24.34 mg of total glucosinolates per gram of dry weight [11], corresponding to an estimated daily glucosinolate intake of 195 mg. Placebo capsules contained 500 mg of cornstarch [21] and were identical in appearance to the intervention capsules. Watercress processing, encapsulation, and packaging were performed by Tungsuwan Organic Farm Co., Ltd., Chiang Mai, Thailand.

### 2.2. Human Ethical Approval

This pilot study was approved by the Human Experimentation Committee (HEC) of the Research Institute for Health Sciences, Chiang Mai University (Approval No. 66/2024; Project No. 22/67) on 10 October 2024. The study protocol was subsequently registered with the Thai Clinical Trials Registry (TCTR), Thailand (https://www.thaiclinicaltrials.org; Registration No. TCTR20251119004) on 19 November 2025. Registration was completed after completion of participant recruitment because the parent trial was originally designed as an institutional pilot study evaluating clinical cardiovascular outcomes rather than a regulatory clinical trial. The retrospective registration represents a limitation of the study.

### 2.3. Participants

Participants were recruited according to predefined inclusion and exclusion criteria. Eligible participants were Thai adults aged 40–59 years with a low-to-moderate cardiovascular disease (CVD) risk, defined as a predicted 10-year Framingham CVD risk of <15%, and at least one cardiometabolic risk factor: systolic blood pressure (SBP) of 130–160 mmHg, total cholesterol (TC) of 200–240 mg/dL, fasting blood glucose (FBG) of 100–125 mg/dL, body mass index (BMI) of 25–40 kg/m^2^, or waist circumference >90 cm in men or >80 cm in women [22]. Individuals with chronic medical or psychiatric conditions requiring pharmacological treatment, as well as pregnant or lactating women, were excluded. Before enrollment, all eligible participants received a detailed explanation of the study objectives and procedures and provided written informed consent.

### 2.4. Sample Size

The sample size was determined during the design of the parent randomized pilot trial [17] using G*Power version 3.1.9.7 (Heinrich Heine University Düsseldorf, Germany). The calculation was based on comparing two independent means using the previously reported between-group difference in HDL-C of 2.7 mg/dL, assuming standard deviations of 2.0 and 2.2 mg/dL for the intervention and placebo groups, respectively. Using a two-sided significance level of 0.05 and 80% statistical power, the minimum required sample size was estimated to be 10 participants per group. To allow for an anticipated 25% attrition rate, the target enrollment was increased to 13 participants per group (26 participants in total). All 26 randomized participants completed both study visits and were included in the metabolomics analysis.

### 2.5. Study Design

This exploratory metabolomics investigation was conducted using archived plasma samples collected from a previously completed randomized, single-blind, placebo-controlled pilot trial which was conducted between January and March 2025. A total of 27 participants were enrolled; however, only 26 met the inclusion criteria. Participants were randomly assigned (1:1) using computer-generated block randomization with allocation concealment through sequentially numbered opaque sealed containers prepared by an independent researcher.

Participants were blinded to treatment allocation throughout the intervention. Watercress and placebo capsules were identical in size, color, weight, packaging, and labeling. Although watercress possesses a characteristic odor, encapsulation minimized perceptible differences, and no participant reported identifying their treatment allocation. Formal assessment of blinding success was not performed.

Investigators responsible for clinical follow-up were aware of treatment allocation because of the single-blind study design. However, plasma samples submitted for ^1^H-NMR acquisition were anonymized using unique study identification numbers before laboratory analysis. The NMR operators and metabolomics analysts were therefore blinded to treatment allocation during spectral acquisition, metabolite identification, and statistical analyses.

Participants were instructed to maintain their habitual diet, physical activity, and lifestyle throughout the intervention. Consumption of additional cruciferous vegetables or nutritional supplements was discouraged during the study period. Dietary adherence and compliance were monitored twice weekly by telephone, although detailed dietary intake was not quantified using food records or dietary recalls.

**Figure 1:**
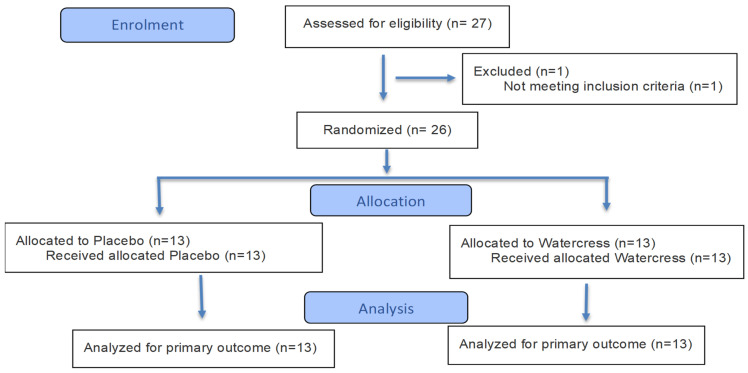
Participant flow diagram for the randomized, single-blind, placebo-controlled study.

Clinical assessments and fasting blood samples were obtained at baseline (Day 0) and after 28 days of intervention. Plasma was separated from heparinized venous blood by centrifugation at 3000× *g* for 10 min, aliquoted, and stored at −80 °C until metabolomic analysis. All the participants completed the study (Figure 1).

### 2.6. Preparation of Plasma Samples

All plasma samples were stored at −80 °C until analysis, and repeated freeze–thaw cycles were avoided to preserve sample integrity. Untargeted metabolomic profiling was performed using ^1^H-NMR spectroscopy according to the protocol described by Jaikang et al. [23]. Briefly, plasma samples were lyophilized and reconstituted in 0.6 mL of 0.1 µM trimethylsilylpropionic acid (TSP) prepared in deuterium oxide (D_2_O), which served as the internal chemical shift reference. The prepared samples were transferred into 5 mm NMR tubes and analyzed on a 500 MHz NMR spectrometer using water suppression to minimize the residual water signal. All samples were processed using reagents of analytic grade from Sigma-Aldrich (St. Louis, MO, USA).

### 2.7. Acquisition of ^1^H-NMR Spectra

^1^H-NMR spectral acquisition was performed according to the protocol described by Jaikang et al. [24]. Briefly, plasma spectra were acquired using a 500 MHz Bruker AVANCE spectrometer (Bruker BioSpin, Rheinstetten, Germany) equipped with a Carr–Purcell–Meiboom–Gill (CPMG, RD–90–(τ–180–τ)^n^–acquire) pulse sequence to suppress broad macromolecular signals. Spectra were acquired at 27 °C using the following parameters: spectral width, 8278.146 Hz; dwell time, 60.40 μs; free induction decay (FID) resolution, 0.126 Hz; relaxation delay, 1 s; acquisition time, 3.95 s; and 16 signal averages (scans) following a 90° excitation pulse. Spectral processing, including Fourier transformation, phase correction, and baseline correction, was performed using TopSpin version 4.0.7 software. Spectra were analyzed over the chemical shift range of 0–12 ppm and normalized to the total spectral integral before downstream metabolomic analyses.

### 2.8. Metabolite Identification by Database and Spectral Analysis

Metabolite identification was performed by matching the acquired ^1^H-NMR spectra with reference data from the Human Metabolome Database (HMDB). Spectral assignment was based on chemical shift positions, J-coupling constants, and characteristic spin–spin coupling patterns. Metabolites were considered confidently identified when the observed chemical shifts matched the corresponding HMDB reference values within a tolerance of ±0.01 ppm [23,25].

### 2.9. Data Processing and Analysis

^1^H-NMR spectra were processed, analyzed, and interpreted using MestReNova software (version 12.0.0; Mestrelab Research, Santiago de Compostela, Spain). Metabolite concentrations were quantified from the integrated peak intensities relative to the trimethylsilylpropionic acid (TSP) internal reference [24] and calculated using Equation (1).

(1) $$I_{A}=\frac{H_{A}\times C_{A}}{H_{B}\times C_{B}}\times I_{B}$$

In this equation, $I_{A}$ represents the integrated signal intensity of the metabolite, whereas $I_{B}$ represents the integrated signal intensity of TSP. $H_{A}$ and $H_{B}$ denote the number of contributing protons in the metabolite and TSP signals, respectively. $C_{A}$ represents the calculated metabolite concentration, and $C_{B}$ denotes the known concentration of TSP (µM).

### 2.10. Statistical Analysis

Statistical analyses were performed using Statistical Package for the Social Sciences (SPSS) Version 22 (SPSS Inc., Chicago, IL, USA). Continuous variables were assessed for normality using the Shapiro–Wilk test. Baseline participant characteristics are presented as mean, median and interquartile range (IQR) for continuous variables and as frequency (percentage) for categorical variables. Between-group differences at baseline were evaluated using the Mann–Whitney U test for continuous variables and the Chi-square test for categorical variables.

The effects of watercress supplementation on circulating lipid biomarkers were evaluated using repeated-measures linear mixed-effects models (LMMs) with restricted maximum likelihood estimation. Participant identification was included as a random intercept to account for within-subject correlations arising from repeated measurements, while treatment group (watercress vs. placebo), study visit (baseline and Day 28), and the Group × Visit interaction were specified as fixed effects. The Group × Visit interaction was considered the primary measure of the intervention effect. Model estimates are reported as regression coefficients (β) with corresponding 95% confidence intervals (95% CI) and two-sided *p*-values. To control for multiple comparisons across biomarkers, *p*-values were adjusted using the Benjamini–Hochberg false discovery rate (FDR) procedure. Statistical significance was defined as a two-sided *p* < 0.05.

### 2.11. Metabolomic Data Analysis

Metabolomic data were processed and analyzed using MetaboAnalyst 6.0 (https://www.metaboanalyst.ca/) accessed on 18 June 2026. Prior to analysis, data quality was assessed by verifying non-negative values and excluding missing values. The dataset was normalized using reference sample, log_2_ transformation, and auto-scaling to reduce technical variation and improve comparability across samples. A combined group–time label partial least squares discriminant analysis (PLS-DA) was performed to characterize differences in metabolomic profiles between the watercress and placebo groups at baseline and after the 4-week intervention. Variable Importance in Projection (VIP) scores derived from the PLS-DA model were used to identify metabolites contributing most strongly to group discrimination, with a VIP score ≥ 1.5 considered indicative of a discriminant metabolite. Metabolites meeting this threshold were subsequently compared between groups using independent-samples *t*-tests to assess whether their relative abundances differed significantly between the watercress and placebo groups. Differential metabolites were annotated using the Human Metabolome Database (HMDB, version 5.0). Metabolic pathway analysis was subsequently performed using the *Homo sapiens* pathway library from the Kyoto Encyclopedia of Genes and Genomes (KEGG). Pathway enrichment and pathway topology analyses were integrated to identify significantly perturbed metabolic pathways, with pathway impact values and adjusted *p*-values used to determine biological significance.

## 3. Results

### 3.1. Participants Demographic Characteristics at Baseline

The baseline demographic characteristics, anthropometric measurements, lipid profile, and vital signs of the study participants are summarized in Table 1. A total of 26 participants were randomized equally to the watercress (*n* = 13) and placebo (*n* = 13) groups. The proportion of female participants was identical in both groups (53.8%). Baseline age, body weight, waist circumference, systolic blood pressure, diastolic blood pressure, pulse rate, and lipid profile were comparable between the two groups, with no statistically significant differences observed (all *p* > 0.05). Specifically, the median age was 48.0 (IQR: 44.0–54.0) years in the watercress group and 46.0 (IQR: 45.0–49.0) years in the placebo group. Median body weight was 66.8 (57.2–68.6) kg and 76.0 (61.8–81.7) kg, while median waist circumference was 83.8 (77.6–89.3) cm and 88.2 (82.7–92.7) cm in the watercress and placebo groups, respectively. None of the participants were taking any documented medications.

### 3.2. Effects of Watercress on Plasma Lipid Profile of the Participants

Repeated-measures linear mixed-effects modelling (LMM) identified differential changes in lipid biomarkers between the watercress and placebo groups from Visit 1 to Visit 2. A significant group × visit interaction was observed for LDL-C (β = 24.54, 95% CI: 10.41–38.66; *p* = 0.001), which remained significant after false discovery rate correction (FDR-*p* = 0.003), reflecting a decrease in the watercress group and an increase in the placebo group over time. TC also showed a nominal group × visit interaction (β = 18.85, 95% CI: 1.70–35.99; *p* = 0.031), characterized by a smaller decrease in the watercress group than in the placebo group; however, this association did not remain significant after FDR correction (FDR-*p* = 0.062). No significant group × visit interactions were observed for HDL-C or triglycerides (Table 2).

### 3.3. Metabolomic Dataset

All 26 randomized participants (placebo, *n* = 13; watercress, *n* = 13) completed the 28-day intervention and contributed paired fasting venous blood samples collected at baseline (Day 0) and end-of-intervention (Day 28). All samples met predefined quality-control criteria and were retained for analysis. Untargeted ^1^H-NMR metabolomic profiling generated high-quality spectra that underwent alignment, normalization, and spectral binning prior to multivariate statistical modelling and metabolic pathway analysis. In total, 209 plasma metabolites were identified and included in the final dataset for downstream analyses.

### 3.4. Discrimination Between the Placebo and Watercress Groups

**Figure 2:**
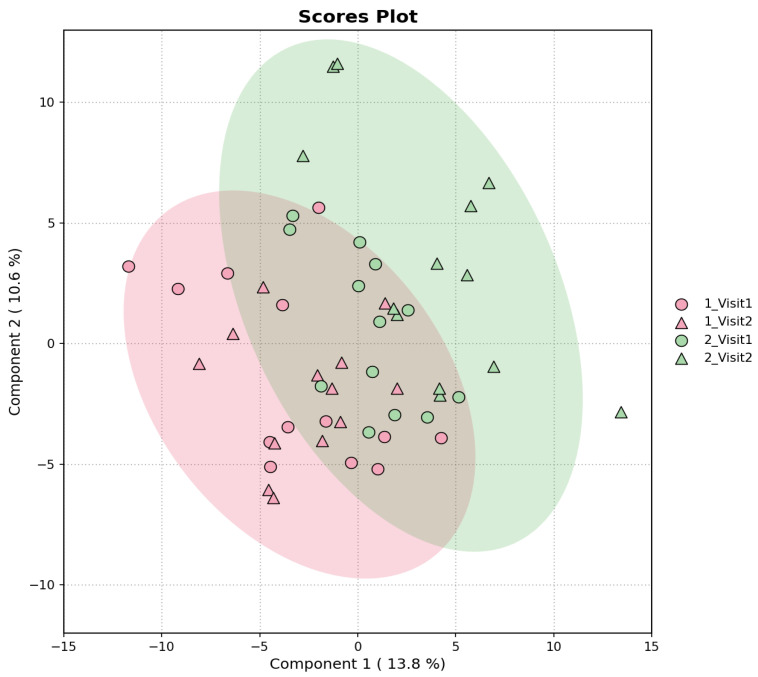
PLS-DA scores plot of plasma ^1^H-NMR metabolomic profiles (combined group-time labels). Group 1 (pink): placebo; Group 2 (green): watercress. Shaded regions denote 95% confidence ellipses. Component 1 and Component 2 explain 13.8% and 10.6% of total variance, respectively. ●: visit 1; ▲: visit 2.

Supervised PLS-DA (combined group-time labels) was performed to investigate metabolic differences between the placebo (Group 1) and watercress (Group 2) groups. The scores plot (Figure 2) demonstrated partial discrimination between the groups along Component 1, which explained 13.8% of the total variance, whereas Component 2 accounted for an additional 10.6%, yielding a cumulative explained variance of 24.4%. The 95% confidence ellipses showed substantial overlap, particularly within the central region of the scores plot, reflecting comparable baseline (visit 1) metabolic profiles and the considerable inter-individual metabolic heterogeneity expected in a small dietary intervention study. Despite this overlap, samples (visit 2) from the watercress group exhibited a consistent shift toward positive Component 1 scores, whereas placebo samples (visit 2) were distributed predominantly toward negative Component 1 values. This directional separation suggests that watercress supplementation induced measurable alterations in the circulating metabolome, although the magnitude of these changes was modest and insufficient to achieve complete group discrimination.

### 3.5. Validation of the PLS-DA Model

**Figure 3:**
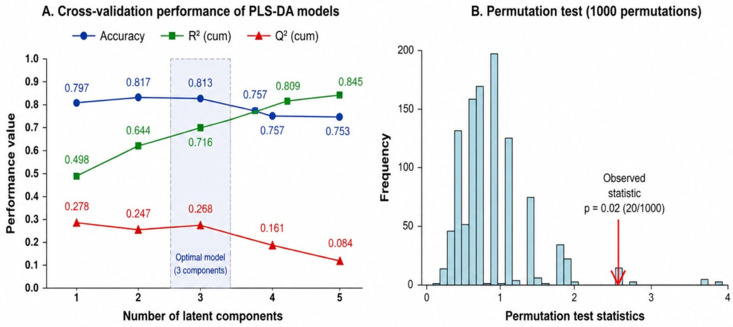
Validation of the PLS-DA model. (**A**) Cross-validation performance of PLS-DA models with one to five latent components showing classification accuracy (blue line), (green line) cumulative explained variance (R^2^), and (red line) cumulative predictive ability (Q^2^). (**B**) Permutation test (1000 permutations) showing the distribution of permuted test statistics. The red arrow indicates the observed test statistic from the original model.

To evaluate the robustness and predictive performance of the combined group-time labels PLS-DA model, cross-validation and permutation testing were performed (Figure 3). Cross-validation of models containing one to five latent components showed that the three-component model provided the optimal balance between model fit and predictive ability, achieving the highest classification accuracy (0.813), with a cumulative explained variance (R^2^) of 0.716 and a cumulative predictive ability (Q^2^) of 0.268 (Figure 3A). Although models with four and five components exhibited higher R^2^ values (0.809 and 0.845, respectively), the marked decline in Q^2^ indicated reduced predictive performance and increasing model complexity, suggesting potential overfitting. Therefore, the three-component model was selected for subsequent analyses. The robustness of the model was further assessed using a permutation test based on 1000 random class permutations (Figure 3B). The observed test statistic lay in the extreme right tail of the null distribution and exceeded the vast majority of permuted models, indicating that the observed class discrimination was unlikely to have occurred by chance (*p* = 0.02; 20/1000 permutations). These validation results demonstrate that the PLS-DA model provides statistically reliable discrimination between the placebo and watercress groups and supports the subsequent interpretation of the discriminant metabolites and pathway analyses.

### 3.6. Discriminant Metabolites (VIP Analysis)

**Figure 4:**
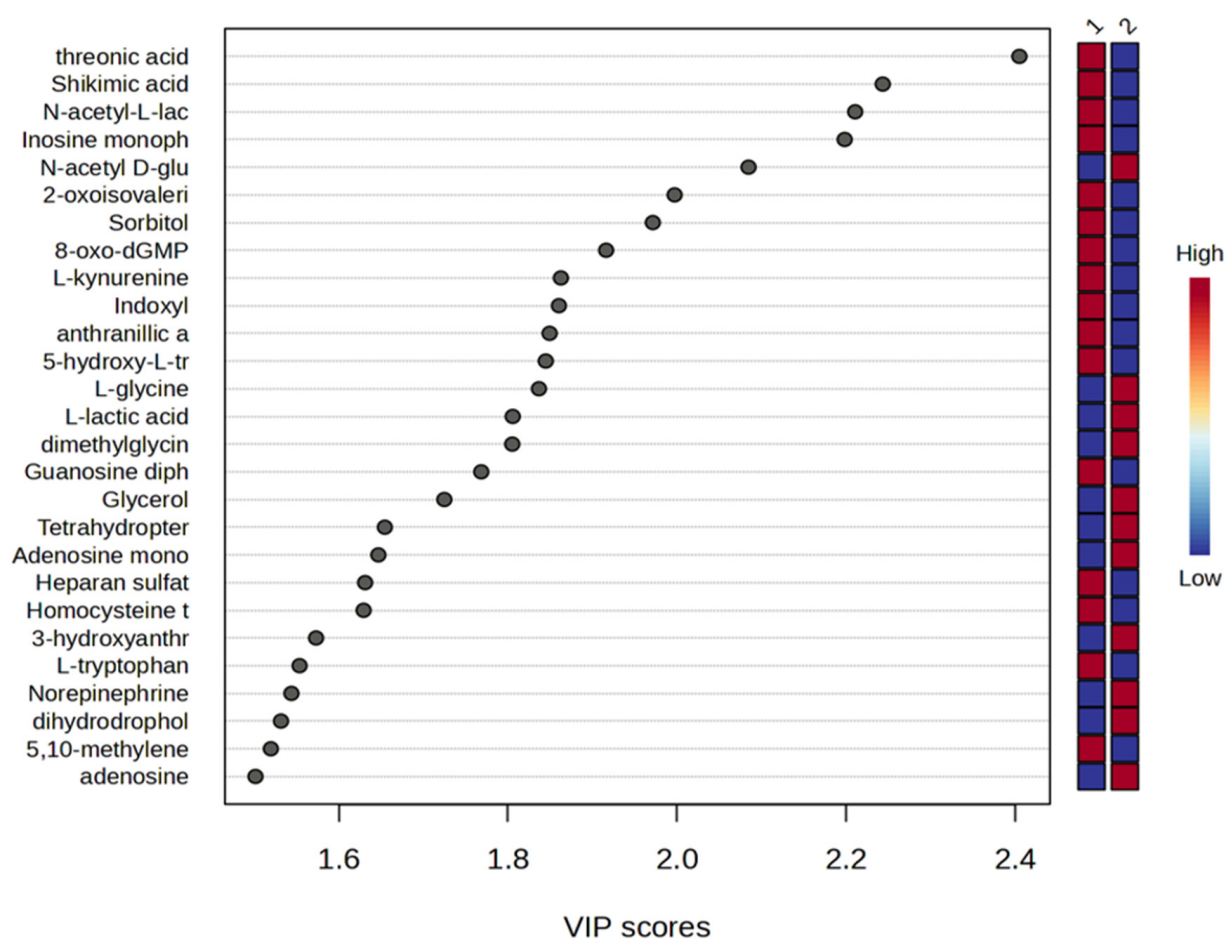
Top discriminant metabolites ranked by combined group-time label PLS-DA variable importance in projection (VIP) score. The adjoining heatmap indicates whether each metabolite was relatively higher (red) or lower (blue) in the placebo (1) and watercress (2) groups. These are relative, semi-quantitative comparisons rather than absolute concentration differences.

The variable importance in projection (VIP) analysis identified 27 metabolites that contributed most strongly to the discrimination between the two study groups, with all selected metabolites exceeding the conventional significance threshold of VIP > 1.5 (Figure 4). VIP scores ranged from approximately 1.50 to 2.41, indicating robust discriminatory power. Threonic acid exhibited the strongest discriminatory contribution, followed by shikimic acid, *N*-acetyl-L-lactate, inosine monophosphate, and *N*-acetyl-D-glutamate. Other highly influential metabolites included 2-oxoisovaleric acid, sorbitol, 8-oxo-dGMP, L-kynurenine, indoxyl sulfate, anthranilic acid, and 5-hydroxy-L-tryptophan. Metabolites associated with amino acid metabolism (L-kynurenine, L-tryptophan, glycine, dimethylglycine, homocysteine thiolactone), nucleotide metabolism (inosine monophosphate, adenosine monophosphate, guanosine diphosphate, adenosine, 8-oxo-dGMP), carbohydrate metabolism (sorbitol, glycerol, L-lactic acid, threonic acid), and microbial co-metabolism (indoxyl sulfate) featured prominently among the discriminating variables. The accompanying heatmap demonstrated distinct abundance patterns between the two groups, with most discriminating metabolites showing reciprocal changes, indicating clear metabolic separation and supporting the robustness of the multivariate model. Collectively, these findings suggest that watercress supplementation induced coordinated alterations across multiple interconnected metabolic pathways, particularly those involved in amino acid, nucleotide, energy, and redox metabolism.

### 3.7. Univariate Analysis of the Discriminant Metabolites

VIP analysis identified 27 discriminant metabolites with VIP scores ≥1.5, of which 19 remained statistically significant after false discovery rate (FDR) correction (FDR-adjusted *p* < 0.05) (Table 3). Threonic acid showed the strongest discrimination (VIP = 2.41; FDR-adjusted *p* = 0.0073), followed by shikimic acid (VIP = 2.25) and *N*-acetyl-L-lactosamine (VIP = 2.21), both with FDR-adjusted *p* = 0.0081. Among the FDR-significant metabolites, decreases were observed in metabolites related to tryptophan metabolism, including L-kynurenine, anthranilic acid, and 5-hydroxy-L-tryptophan, as well as inosine monophosphate, sorbitol, 8-oxo-dGMP, and guanosine diphosphate. In contrast, *N*-acetyl-D-glucosamine, L-glycine, L-lactic acid, dimethylglycine, glycerol, tetrahydropteridine, and adenosine monophosphate were increased. The discriminant metabolite profile indicated alterations across multiple metabolic processes, including tryptophan, carbohydrate and energy, nucleotide, and one-carbon metabolism.

### 3.8. Pathway-Level Analysis

**Figure 5:**
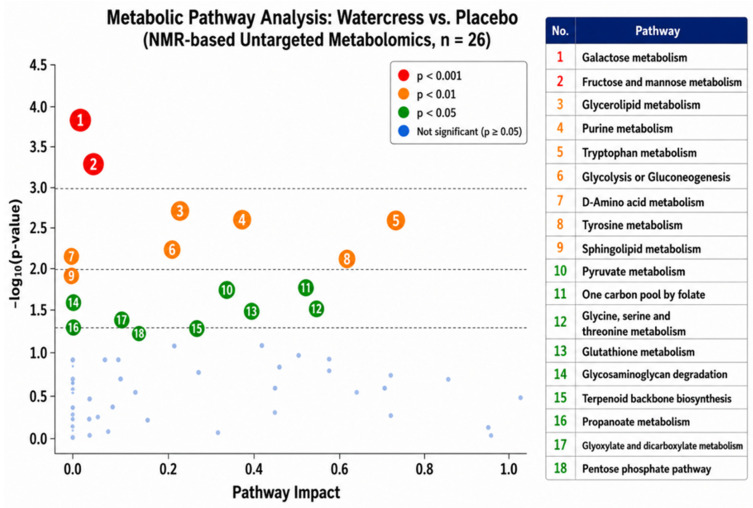
Pathway analysis of placebo and watercress group. Each circle represents an individual metabolic pathway, with the x-axis indicating pathway impact (topological importance) and the y-axis showing statistical significance expressed as −log_10_(*p*-value). Horizontal dashed lines denote significance thresholds corresponding to *p* < 0.05 (−log_10_ = 1.30), *p* < 0.01 (−log_10_ = 2.00), and *p* < 0.001 (−log_10_ = 3.00). Circle colors indicate the level of statistical significance: red, *p* < 0.001; orange, *p* < 0.01; green, *p* < 0.05; and blue, not significant (*p* ≥ 0.05).

Metabolic pathway analysis of the untargeted NMR metabolomics data identified widespread metabolic alterations following 28 days of watercress supplementation compared with placebo. A total of 54 metabolomic pathways were altered following the watercress supplementation (Supplementary Table S1). Out of those 54, eighteen metabolic pathways showed evidence of perturbation, with the magnitude of enrichment represented by pathway impact and statistical significance expressed as −log_10_(*p*-value) (Figure 5). Two pathways surpassed the highly significant threshold (*p* < 0.001), galactose metabolism (Pathway 1) and fructose and mannose metabolism (Pathway 2), indicating that carbohydrate metabolism was the most strongly affected metabolic domain. Six additional pathways were significantly enriched at *p* < 0.01, including glycerolipid metabolism, purine metabolism, tryptophan metabolism, glycolysis/gluconeogenesis, D-amino acid metabolism, and tyrosine metabolism, with pathway impact values ranging from approximately 0.01 to 0.75.

A further ten pathways reached nominal significance (*p* < 0.05), comprising sphingolipid metabolism, pyruvate metabolism, one-carbon metabolism by folate, glycine, serine and threonine metabolism, glutathione metabolism, glycosaminoglycan degradation, terpenoid backbone biosynthesis, propanoate metabolism, glyoxylate and dicarboxylate metabolism, and the pentose phosphate pathway. Among the significantly enriched pathways, purine metabolism and glycolysis/gluconeogenesis exhibited the highest pathway impact values, whereas galactose metabolism demonstrated the greatest statistical significance. These findings suggest that watercress supplementation primarily modulated interconnected pathways involved in carbohydrate utilization, energy metabolism, amino acid metabolism, lipid metabolism, redox homeostasis, and nucleotide metabolism, consistent with broad metabolic remodeling following the intervention.

## 4. Discussion

Four weeks of dried watercress supplementation was associated with detectable changes in the circulating metabolome of middle-aged Thai adults with low-to-moderate cardiovascular risk, despite modest changes in conventional clinical biomarkers. PLS-DA showed partial group separation with modest predictive performance (Q^2^ = 0.268), consistent with subtle metabolic effects in a small, short-term dietary intervention. Nevertheless, 27 discriminant metabolites with VIP scores >1.5 were identified, of which 19 remained statistically significant after false discovery rate correction of univariate *t*-tests. Pathway analysis further identified 18 enriched metabolic pathways, predominantly involving carbohydrate and energy metabolism, tryptophan metabolism, purine metabolism, glycerolipid metabolism, and redox-related pathways. These findings suggest coordinated metabolic adaptations that may precede measurable clinical responses. However, given the pilot design and exploratory analysis, the findings should be considered hypothesis-generating rather than confirmatory.

### 4.1. Carbohydrate and Energy Metabolism

Galactose and fructose/mannose metabolism were among the most significantly perturbed pathways (*p* < 0.001), accompanied by alterations in glycolysis/gluconeogenesis, pyruvate metabolism, and the pentose phosphate pathway (PPP). These pathways are central to carbohydrate utilization, linking dietary sugar metabolism with glycolytic energy production and cellular redox homeostasis [26]. Reduced sorbitol and increased glycerol and L-lactic acid suggest possible changes in polyol pathway activity, glycolytic flux, and lipid turnover. Because fasting glucose and insulin sensitivity were not assessed, these findings cannot establish improved glycemic regulation. The enrichment of the PPP may also be relevant to redox homeostasis through NADPH-dependent glutathione regeneration and could complement Nrf2-mediated antioxidant responses to watercress-derived isothiocyanates [27,28]. Nevertheless, these interpretations require confirmation using targeted metabolic and glycemic measurements.

### 4.2. Amino Acid Metabolism, the Kynurenine Pathway, and One-Carbon Metabolism

Alterations in L-kynurenine, L-tryptophan, anthranilic acid, 5-hydroxy-L-tryptophan, and 3-hydroxyanthranilic acid suggest modulation of the tryptophan–kynurenine pathway, an important regulator of inflammatory and immunometabolic processes. Chronic activation of this pathway, particularly through indoleamine 2,3-dioxygenase 1 (IDO1), has been associated with oxidative stress, endothelial dysfunction, and cardiovascular disease [29]. Although inflammatory biomarkers were not measured, the observed metabolite pattern generates the hypothesis that watercress may influence tryptophan metabolism, potentially consistent with the anti-inflammatory and Nrf2-activating properties of watercress-derived isothiocyanates [30].

Concurrent alterations in glycine, dimethylglycine, homocysteine thiolactone, and 5,10-methylenetetrahydrofolate further suggest modulation of one-carbon metabolism. Increased glycine and dimethylglycine together with reduced homocysteine thiolactone may reflect changes in glutathione synthesis, methyl-group metabolism, and redox regulation [31,32]. These findings identify interconnected tryptophan and one-carbon metabolic networks as candidate mechanisms for future targeted investigation.

### 4.3. Nucleotide Metabolism and Oxidative DNA Damage

Purine metabolism was among the most strongly perturbed pathways, with alterations in inosine monophosphate, adenosine monophosphate, guanosine diphosphate, adenosine, and 8-oxo-dGMP. Reduced 8-oxo-dGMP, a marker of oxidative DNA damage [33], is consistent with previously reported antioxidant effects of watercress and the Nrf2-activating properties of PEITC. Changes in purine nucleotides may additionally reflect altered nucleotide turnover and cellular energy homeostasis [34]. Increased adenosine is also of potential vascular relevance because of its vasodilatory and anti-inflammatory actions [29], although its relationship with clinical outcomes in this study cannot be established from the present data and requires confirmation in targeted functional studies.

### 4.4. Microbial Co-Metabolism and Gut–Host Interactions

Reductions in indoxyl sulfate and shikimic acid suggest potential alterations in gut–host co-metabolism. Indoxyl sulfate, a microbiota-derived product of tryptophan metabolism, has been associated with endothelial dysfunction, oxidative stress, and cardiovascular risk [35], whereas shikimic acid is linked to microbial aromatic amino acid metabolism and has reported anti-inflammatory and intestinal barrier-protective properties through inhibition of MAPK and NF-κB signaling and suppression of pro-inflammatory cytokines [36,37]. However, without microbiome profiling or detailed dietary assessment, the relative contributions of microbial and host metabolism cannot be distinguished. These findings therefore generate a testable hypothesis that watercress may influence gut microbial metabolism, requiring confirmation through integrated microbiome and targeted metabolomic studies.

### 4.5. Lipid Metabolism and Clinical Biomarkers

Alterations in glycerolipid and sphingolipid metabolism suggest potential effects of watercress supplementation on lipid homeostasis beyond conventional lipid measures. Increased glycerol may reflect altered glycerolipid turnover, while sphingolipid perturbations are potentially relevant given the roles of ceramides and related metabolites in inflammation, insulin resistance, and atherosclerosis [32,38]. These findings align with the significant Group × Visit interaction for LDL-C, which remained significant after multiple-testing correction and reflected decreased LDL-C in the watercress group but increased levels in the placebo group. These findings suggest potential modulation of lipid metabolism; however, they remain exploratory and require confirmation in larger studies incorporating lipoprotein subfraction and apolipoprotein measurements.

### 4.6. Redox Homeostasis and Glutathione Metabolism

The nominal enrichment of glutathione metabolism together with increased glycine, reduced homocysteine thiolactone, and reduction in 8-oxo-dGMP suggest modulation of antioxidant and redox-related pathways. Glycine is a precursor of glutathione, while reduced homocysteine thiolactone may reflect altered one-carbon and redox metabolism [32]. These changes are biologically consistent with PEITC, a major watercress-derived isothiocyanate capable of activating Nrf2-regulated antioxidant and detoxification pathways [39]. Alterations in threonic acid, a metabolite of vitamin C, further support potential changes in antioxidant metabolism [40]. However, these metabolomic signatures do not directly demonstrate enhanced antioxidant capacity and should be validated using targeted biochemical measurements.

Previous watercress interventions have primarily examined predefined outcomes such as blood pressure, lipid profiles, oxidized LDL, antioxidant capacity, and DNA damage. The present untargeted metabolomic analysis extends this evidence by identifying candidate alterations across interconnected pathways involving energy metabolism, tryptophan metabolism, nucleotide turnover, redox regulation, lipid metabolism, and gut–host co-metabolism. This multi-pathway response is biologically plausible given the complex phytochemical composition of watercress [41,42], but requires validation in larger trials using targeted metabolomics, complementary multi-omics approaches, and pathway-specific clinical biomarkers.

### 4.7. Study Limitations

Several limitations should be considered when interpreting these findings. First, the small sample size (*n* = 26), inherent to the pilot design, limited statistical power and may have reduced the ability to detect modest metabolic and clinical effects. Second, the 4-week intervention period may have been insufficient to produce substantial changes in conventional cardiometabolic biomarkers, despite detectable alterations in the circulating metabolome. Third, although untargeted ^1^H-NMR metabolomics provides reproducible and broad metabolic profiling, it has lower analytical sensitivity than mass spectrometry-based platforms, and the resulting metabolite measurements are predominantly semi-quantitative. Fourth, the modest predictive performance of the PLS-DA model warrants cautious interpretation, and the identified discriminant metabolites require confirmation through targeted quantitative analyses and validation in independent cohorts. Fifth, dietary intake, gut microbiome composition, and circulating inflammatory mediators were not comprehensively characterized, limiting the ability to directly link the observed metabolic alterations to specific biological mechanisms. Finally, the parent trial was retrospectively registered, and the effectiveness of participant blinding was not formally assessed; these factors should be considered when interpreting the findings.

Future studies should employ adequately powered randomized controlled trials with longer intervention and follow-up periods, targeted quantitative validation of key metabolites, and integration of complementary multi-omics approaches, including metabolomics, proteomics, lipidomics, and microbiome profiling. Concurrent assessment of dietary intake, inflammatory and endothelial biomarkers, and cardiovascular outcomes would further enable evaluation of relationships between watercress-induced metabolic changes and physiological responses. Such studies will be important for validating the metabolic signatures identified in this analysis and determining their potential mechanistic and clinical relevance.

## 5. Conclusions

This randomized, single-blind, placebo-controlled pilot study provides an untargeted ^1^H-NMR metabolomic characterization of the systemic metabolic response to watercress supplementation in middle-aged Thai adults with low-to-moderate cardiovascular risk. Four weeks of supplementation were associated with candidate alterations across multiple metabolic pathways, including carbohydrate and energy metabolism, tryptophan-kynurenine metabolism, purine metabolism, one-carbon metabolism, glutathione metabolism, lipid metabolism, and gut microbial co-metabolism. Changes in metabolites related to oxidative stress, inflammatory signaling, redox homeostasis, and microbial metabolism, including indoxyl sulfate and shikimic acid, provide potential biological leads from which hypotheses regarding the cardiometabolic effects of watercress can be generated. However, these pathway-level interpretations should not be considered evidence of antioxidant, anti-inflammatory, or other causal mechanisms, as the corresponding biological processes were not directly assessed. The observed clinical and metabolomic patterns therefore provide complementary, but not mechanistically linked, signals that warrant further investigation. Given the small sample size, modest predictive performance of the PLS-DA model, semi-quantitative nature of untargeted ^1^H-NMR metabolomics, and short intervention duration, these findings should be regarded as preliminary and hypothesis-generating rather than confirmatory. Larger, prospectively registered and adequately powered randomized controlled trials incorporating targeted metabolite quantification, complementary multi-omics approaches, microbiome profiling, and relevant cardiovascular, inflammatory, and endothelial biomarkers are required to validate these candidate metabolic signatures and test the biological hypotheses generated by the present study.

## Figures and Tables

**Table 1:** Baseline demographics and vital signs (*n* = 26).

Variable	Watercress (*n* = 13)	Placebo (*n* = 13)	*p*-Value
**Female, *n* (%)**	7 (53.8)	7 (53.8)	1.000
**Age, years**	48.0 (44.0–54.0)	46.0 (45.0–49.0)	0.455
**Weight, kg**	66.8 (57.2–68.6)	76.0 (61.8–81.7)	0.259
**Waist, cm**	83.8 (77.6–89.3)	88.2 (82.7–92.7)	0.505
**SBP, mmHg**	125.0 (120.0–153.0)	130.0 (125.0–137.0)	0.837
**DBP, mmHg**	80.0 (75.0–95.0)	86.0 (79.0–92.0)	0.555
**Pulse, bpm**	70.0 (63.0–84.0)	73.0 (69.0–74.0)	0.878
**TC**	241.1 ± 54.5	226.2 ± 51.5	0.365
**HDL-C**	46.5 ± 6.0	49.2 ± 6.8	0.472
**LDL-C**	163.7 ± 47.1	163.2 ± 45.1	0.783
**TG**	159.4 ± 92.5	108.2 ± 35.1	0.087

Note: *n*: sample size, cm: centimeters; kg: kilograms; mmHg: millimeters of mercury; bpm: beats per minute; HDL-C: high-density lipoprotein cholesterol; LDL-C: low-density lipoprotein cholesterol; TC: total cholesterol; TG: triglycerides.

**Table 2:** LMM analysis of lipid profile of the participants (*n* = 26).

Biomarker	Watercress V1	Watercress V2	Placebo V1	Placebo V2	Group × Visit β (95% CI)	*p*-Value	FDR-*p* Value
TC	241.1 ± 54.5	233.8 ± 53.5	226.2 ± 51.5	200.0 ± 36.2	18.85 (1.70 to 35.99)	0.031 *	0.062
HDL-C	46.5 ± 6.0	47.6 ± 6.1	49.2 ± 6.8	47.1 ± 7.5	3.23 (−0.19 to 6.65)	0.064	0.085
LDL-C	163.7 ± 47.1	155.2 ± 52.9	163.2 ± 45.1	180.2 ± 38.6	24.54 (10.41 to 38.66)	0.001 *	0.003 *
TG	159.4 ± 92.5	152.2 ± 135.7	108.2 ± 35.1	109.0 ± 51.6	−7.92 (−52.07 to 36.22)	0.725	0.72

Values are presented as mean ± SD (standard deviation). Repeated measures linear mixed-effects models (LMM) were used to assess changes over time, with participant included as a random effect and group, visit, and group × visit interaction as fixed effects. The group × visit β coefficient (95% CI) represents the difference between groups in the change from Visit 1 (V1; baseline) to Visit 2 (V2; post-intervention). *p*-values correspond to the group × visit interaction and were adjusted for multiple comparisons using the false discovery rate. FDR: false discovery rate; HDL-C: high-density lipoprotein cholesterol; LDL-C: low-density lipoprotein cholesterol; TC: total cholesterol; TG: triglycerides; V1: visit one (Day 0); V2: visit two (Day 28). * Statistically significant at *p* < 0.05.

**Table 3:** PLS-DA-Derived Discriminant Plasma Metabolites and Their Univariate Statistical Significance Following Watercress Supplementation.

Metabolite	VIP Score	Direction of Change (1 → 2)	*t*-Statistic	*p*-Value	−log_10_(*p*)	FDR-Adjusted *p*
Threonic acid	2.41	↓	−4.142	3.47 × 10^−5^ *	4.460	0.0073 *
Shikimic acid	2.25	↓	−3.864	1.12 × 10^−4^ *	3.950	0.0081 *
*N*-acetyl-L-lactate	2.21	↓	−3.808	1.41 × 10^−4^ *	3.851	0.0081 *
Inosine monophosphate	2.20	↓	−3.786	1.54 × 10^−4^ *	3.813	0.0081 *
*N*-acetyl-D-glucosamine	2.09	↑	3.591	3.31 × 10^−4^ *	3.480	0.0139 *
2-Oxoisovaleric acid	2.00	↓	−3.440	5.84 × 10^−4^ *	3.234	0.0204 *
Sorbitol	1.97	↓	−3.396	6.87 × 10^−4^ *	3.163	0.0206 *
8-oxo-dGMP	1.91	↓	−3.301	9.68 × 10^−4^ *	3.014	0.0252 *
L-Kynurenine	1.87	↓	−3.208	0.00134 *	2.873	0.0252 *
Indoxyl	1.87	↓	−3.204	0.00136 *	2.867	0.0252 *
Anthranillic acid	1.86	↓	−3.185	0.00145 *	2.839	0.0252 *
5-Hydroxy-L-tryptophan	1.86	↓	−3.178	0.00149 *	2.827	0.0252 *
L-Glycine	1.85	↑	3.164	0.00156 *	2.806	0.0252 *
L-Lactic acid	1.81	↑	3.110	0.00187 *	2.727	0.0263 *
Dimethylglycine	1.81	↑	3.109	0.00188 *	2.726	0.0263 *
Guanosine diphosphate	1.77	↓	−3.046	0.00232 *	2.634	0.0305 *
Glycerol	1.72	↑	2.971	0.00298 *	2.526	0.0368 *
Tetrahydropterin	1.66	↑	2.850	0.00438 *	2.358	0.0370 *
Adenosine monophosphate	1.65	↑	2.837	0.00457 *	2.340	0.0400 *
Heparan sulfate	1.63	↓	−2.810	0.00497 *	2.304	0.0502
Homocysteine thiolactone	1.63	↓	−2.807	0.00502 *	2.300	0.0502
3-Hydroxyanthranilic acid	1.57	↑	2.710	0.00675 *	2.171	0.0644
L-Tryptophan	1.55	↓	−2.676	0.00746 *	2.127	0.0681
Norepinephrine	1.54	↑	2.660	0.00784 *	2.106	0.0686
Dihydrobiopterin	1.53	↑	2.638	0.00835 *	2.079	0.0701
5,10-Methylenetetrahydrofolate	1.52	↓	−2.618	0.00887 *	2.052	0.0716
Adenosine	1.50	↑	2.586	0.00972 *	2.013	0.0722

Univariate statistical analysis of plasma metabolites associated with watercress supplementation after 28 days of intervention. Metabolites are ranked according to increasing *p*-value obtained from independent *t*-tests comparing the watercress and placebo groups. Positive *t*-statistics indicate higher relative metabolite abundance in the watercress group, whereas negative *t*-statistics indicate lower abundance. *p*-values were adjusted for multiple comparisons using the Benjamini–Hochberg false discovery rate (FDR). Procedure arrows indicate changes from Group 1 (Placebo) to Group 2 (Watercress) (↑ increased; ↓ decreased). VIP (Variable Importance in Projection) scores were obtained from the combined group-time labels PLS-DA model. * Significant at *p* < 0.05. These are relative, semi-quantitative comparisons rather than absolute concentration differences.

## Data Availability

The original contributions presented in this study are included in the article/Supplementary Materials. Further inquiries can be directed to the corresponding author.

## Supplementary Materials

The following supporting information can be downloaded at https://www.mdpi.com/article/10.3390/nu18152559/s1, Table S1: Altered pathways following watercress supplementation.

## Abbreviations

The following abbreviations are used in this manuscript:

^1^H-NMR	Proton Nuclear Magnetic Resonance
8-oxo-dGMP	8-Oxo-2′-deoxyguanosine monophosphate
AMP	Adenosine Monophosphate
BMI	Body Mass Index
CI	Confidence Interval
CPMG	Carr–Purcell–Meiboom–Gill
CVD	Cardiovascular Disease
D_2_O	Deuterium Oxide
DBP	Diastolic Blood Pressure
FBG	Fasting Blood Glucose
FDR	False Discovery Rate
GST	Glutathione S-Transferase
HDL-C	High-Density Lipoprotein Cholesterol
HMDB	Human Metabolome Database
IDO1	Indoleamine 2,3-Dioxygenase 1
IQR	Interquartile Range
KEGG	Kyoto Encyclopedia of Genes and Genomes
LDL-C	Low-Density Lipoprotein Cholesterol
LMM	Linear Mixed-Effects Model
MAPK	Mitogen-activated protein kinase
Nrf2	Nuclear Factor Erythroid 2-Related Factor 2
PEITC	Phenethyl Isothiocyanate
PLS-DA	Partial Least Squares-Discriminant Analysis
PPP	Pentose Phosphate Pathway
SBP	Systolic Blood Pressure
SD	Standard Deviation
SPSS	Statistical Package for the Social Sciences
TC	Total Cholesterol
TCTR	Thai Clinical Trials Registry
TG	Triglycerides
TSP	Trimethylsilylpropionic Acid
VIP	Variable Importance in Projection

## References

[1] Tan S.C.W., Zheng B.-B., Tang M.-L., Chu H., Zhao Y.-T., Weng C. (2025). Global burden of cardiovascular diseases and its risk factors, 1990–2021: A systematic analysis for the global burden of disease study 2021. QJM Int. J. Med..

[2] Bhoothookngoen P., Sanchan N. (2024). Prevalence of noncommunicable diseases and social determinants of health in Thailand: Insights from public datasets. Thai J. Public Health.

[3] Suanrueang P. (2024). A comparison of the disease occurrence of cerebrovascular diseases, diabetes mellitus, hypertensive diseases, and ischaemic heart diseases among hospitalized older adults in Thailand. Sci. Rep..

[4] Tangjittipokin W., Srisawat L., Teerawattanapong N., Narkdontri T., Homsanit M., Plengvidhya N. (2022). Prevalence and characteristics of prediabetes and metabolic syndrome in seemingly healthy persons at a Health check-up clinic. J. Multidiscip. Healthc..

[5] Kucia A.M., Hartley A. (2022). Risk factors for cardiovascular disease. Card. Care A Pract. Guide Nurses.

[6] Xie C., Yuan Y., Wang Y., Qi C., Wang W., An C., Aikepaer A., Zhang Y., Zhang G., Feng X. (2025). Beyond Discrete Diagnoses: Conceptualizing Obesity-associated Metabolic Disorders as a Unified, Dynamic Continuum. Curr. Obes. Rep..

[7] Alanzi S.S., Almayouf S.M.A., Alotaibi K.M.N., Alanazi A.O.A., Ghuwayzi H., Alharbi O.N., Aldajani O.H.O. (2025). Global burden, risk factors, and evidence-based strategies for cardiovascular disease prevention: A comprehensive systematic review. J. Angiother..

[8] Samuel P.O., Edo G.I., Emakpor O.L., Oloni G.O., Ezekiel G.O., Essaghah A.E.A., Agoh E., Agbo J.J. (2024). Lifestyle modifications for preventing and managing cardiovascular diseases. Sport Sci. Health.

[9] Chaudhari V., Singh O.B., Gouthami N., Thakur N., Singh R., Singh S., Thapa U., Nagar B.L. (2024). Unlocking the nutritional power of vegetables: A guide to vibrant health. Eur. J. Nutr. Food Saf..

[10] Seferli M., Adamantidi T., Tsoupras A. (2026). Aquatic Plants: Bioactive Profile, Extraction Methods, and Health-Promoting Properties for Applications in the Food, Cosmetic, and Pharmaceutical Industries. Natural Products: Phytochemistry, Botany, Metabolism of Alkaloids, Phenolics and Terpenes.

[11] Kijkuokool P., Stepanov I., Ounjaijean S., Koonyosying P., Rerkasem K., Chuljerm H., Parklak W., Kulprachakarn K. (2024). Effects of drying methods on the phytochemical contents, antioxidant properties, and anti-diabetic activity of *Nasturtium officinale* R. Br. (Betong watercress) from Southern Thailand. Life.

[12] Wen J., Karabala M., Muttalib Z., Syed B., Khalil R., Razick D., Razick A., Pai D. (2025). Evaluating the Anti-Oxidant and Anti-Inflammatory Properties of Watercress Supplementation at Short-Term Follow-Up: A Systematic Review of Randomized Controlled Trials. Food Sci. Nutr..

[13] Baldelli S., Lombardo M., D’Amato A., Karav S., Tripodi G., Aiello G. (2025). Glucosinolates in human health: Metabolic pathways, bioavailability, and potential in chronic disease prevention. Foods.

[14] Kyriakou S., Michailidou K., Amery T., Stewart K., Winyard P.G., Trafalis D.T., Franco R., Pappa A., Panayiotidis M.I. (2022). Polyphenolics, glucosinolates and isothiocyanates profiling of aerial parts of *Nasturtium officinale* (Watercress). Front. Plant Sci..

[15] Hecht S.S., Carmella S.G., Basu S., Chen M., Wittman M., Tang M.K., Zhao Y., Lu J., Ng D., Heath E. (2026). A watercress drink increases detoxification of common environmental carcinogens such as acrolein and benzene: Results of a randomized clinical trial. Carcinogenesis.

[16] Gill C.I., Haldar S., Boyd L.A., Bennett R., Whiteford J., Butler M., Pearson J.R., Bradbury I., Rowland I.R. (2007). Watercress supplementation in diet reduces lymphocyte DNA damage and alters blood antioxidant status in healthy adults. Am. J. Clin. Nutr..

[17] Kijkuokool P., Rerkasem K., Fakfum P., Parklak W., Chuljerm H., Khiaolaongam W., Maluwa C., Kulprachakarn K. (2026). Efficacy of Watercress (*Nasturtium officinale* R. Br.) Consumption on Blood Pressure, Oxidative Stress Biomarkers, and Estimated Cardiovascular Risk in Thai Middle-Aged Adults: A Randomized Placebo-Controlled Pilot Study. Antioxidants.

[18] Di Paco G., Meoni G., Ghini V., Vignoli A., Tenori L., Turano P., Luchinat C. (2026). Recent trends in metabolomics by NMR spectroscopy. Angew. Chem. Int. Ed..

[19] Kotwal S., Shin J.W., Bezabeh T. (2025). Predicting and monitoring treatment response via 1-H NMR metabolomics of plasma, serum, and urine. Biophys. Rev..

[20] Takis P.G., Aggelidou V.A., Sands C.J., Louka A. (2023). Mapping of 1H NMR chemical shifts relationship with chemical similarities for the acceleration of metabolic profiling: Application on blood products. Magn. Reson. Chem..

[21] Quamri M.A., Samal M., Alam M.A. (2026). Efficacy of Jadwar (*Delphinium denudatum* Wall. ex Hook. f. & Thomson) in Subclinical Hypothyroid Patients: A Single-Blind, Randomized Placebo Controlled Trial. Phytomedicine.

[22] de Menezes-Júnior L.A.A., de Moura S.S., Carraro J.C.C., de Freitas S.N., Pimenta F.A.P., Machado-Coelho G.L.L., de Oliveira F.L.P., do Nascimento Neto R.M., Meireles A.L. (2025). Framingham score adapted: A valid alternative for estimating cardiovascular risk in epidemiological studies. BMC Cardiovasc. Disord..

[23] Jaikang C., Konguthaithip G., Amornlertwatana Y., Autsavapromporn N., Rattanachitthawat S., Monum T. (2024). Alterations in the blood kynurenine pathway following long-term PM2. 5 and PM10 exposure: A cross-sectional study. Biomedicines.

[24] Jaikang C., Konguthaithip G., Amornlertwatana Y., Autsavapromporn N., Rattanachitthawat S., Liampongsabuddhi N., Monum T. (2025). Metabolic Disruptions and Non-Communicable Disease Risks Associated with Long-Term Particulate Matter Exposure in Northern Thailand: An NMR-Based Metabolomics Study. Biomedicines.

[25] Madrid-Gambin F., Oller S., Marco S., Pozo Ó.J., Andres-Lacueva C., Llorach R. (2023). Quantitative plasma profiling by 1H NMR-based metabolomics: Impact of sample treatment. Front. Mol. Biosci..

[26] Mir F.A., Ullah E., Mall R., Iskandarani A., Samra T.A., Cyprian F., Parray A., Alkasem M., Abdalhakam I., Farooq F. (2022). Dysregulated metabolic pathways in subjects with obesity and metabolic syndrome. Int. J. Mol. Sci..

[27] Lee W.D., Weilandt D.R., Liang L., MacArthur M.R., Jaiswal N., Ong O., Mann C.G., Chu Q., Hunter C.J., Ryseck R.-P. (2025). Lactate homeostasis is maintained through regulation of glycolysis and lipolysis. Cell Metab..

[28] TeSlaa T., Ralser M., Fan J., Rabinowitz J.D. (2023). The pentose phosphate pathway in health and disease. Nat. Metab..

[29] Chen W., Shui M., Wei Z., Gao W., Liu X., Lei Q. (2026). The tryptophan-kynurenine pathway in cardiovascular diseases: Mechanistic insights and therapeutic opportunities. Front. Cardiovasc. Med..

[30] Ahmed N.A., Allam A.A., Rudayni H.A., Alshabrmi F.M., Alkhayl F.F.A., Abdelrheem D.A., Lamsabhi A.M., Othman S.I., Kamel E.M. (2025). Watercress-derived glucosinolates as potential allosteric PTP1B inhibitors: A dual in silico and in vitro study on insulin signaling modulation. BMC Chem..

[31] Dang S., Jain A., Dhanda G., Bhattacharya N., Bhattacharya A., Senapati S. (2024). One carbon metabolism and its implication in health and immune functions. Cell Biochem. Funct..

[32] Flori L., Veneziano S., Martelli A., Piragine E., Calderone V. (2025). Transsulfuration Pathway Products and H2S-Donors in Hyperhomocysteinemia: Potential Strategies Beyond Folic Acid. Int. J. Mol. Sci..

[33] Zhong Y., Zhang X., Feng R., Fan Y., Zhang Z., Zhang Q.W., Wan J.B., Wang Y., Yu H., Li G. (2024). OGG1: An emerging multifunctional therapeutic target for the treatment of diseases caused by oxidative DNA damage. Med. Res. Rev..

[34] Lopez-Schenk R., Collins N.L., Schenk N.A., Beard D.A. (2024). Integrated functions of cardiac energetics, mechanics, and purine nucleotide metabolism. Compr. Physiol..

[35] Zhang Y., Han X., Feng T., Li Z., Yu H., Chen Y., Gao Y., Gao Q., Zhang L., Li S. (2025). Gut-microbiota-derived indole sulfate promotes heart failure in chronic kidney disease. Cell Host Microbe.

[36] Gandhi G.R., Vasconcelos A.B.S., Antony P.J., Montalvão M.M., de Franca M.N.F., Hillary V.E., Ceasar S.A., Liu D. (2023). Natural sources, biosynthesis, biological functions, and molecular mechanisms of shikimic acid and its derivatives. Asian Pac. J. Trop. Biomed..

[37] Li X., Mo K., Tian G., Zhou J., Gong J., Li L., Huang X. (2023). Shikimic acid regulates the NF-κB/MAPK signaling pathway and gut microbiota to ameliorate DSS-induced ulcerative colitis. J. Agric. Food Chem..

[38] Delcheva G., Stefanova K., Stankova T. (2024). Ceramides—Emerging biomarkers of lipotoxicity in obesity, diabetes, cardiovascular diseases, and inflammation. Diseases.

[39] Kamal R.M., Abdull Razis A.F., Mohd Sukri N.S., Perimal E.K., Ahmad H., Patrick R., Djedaini-Pilard F., Mazzon E., Rigaud S. (2022). Beneficial health effects of glucosinolates-derived isothiocyanates on cardiovascular and neurodegenerative diseases. Molecules.

[40] Kumar A., Mehan S., Gupta S., Gupta G.D., Samant R., Tongra M. (2026). Enhanced neurophysiological benefits of magnesium-acetyl-taurate over magnesium-l-threonate: A comparative pre-clinical study on bioavailability, synaptic plasticity and cognitive functions. NeuroMol. Med..

[41] Sengupta B., Fisher A., Villanueva J., Simington L., Jenkins B., Travis K., Roy D. (2025). Analysis of phytochemicals in watercress leaves using chromatographic, spectroscopic and in-vitro approaches. Results Chem..

[42] Riaz T., Azhar A., Xia Z., Batool A., Ye X., Khalid B., Khan M.M., Riaz R., Hayat S., Butt M.A. (2026). Advances in plant-based functional foods: Emerging trends, nutritional potential, and health implications. Food Sci. Appl. Microbiol. Rep..

