# Supplementary material for: Untargeted 1H-NMR Metabolomics Identifies Candidate Metabolic Changes Associated with Watercress (Nasturtium officinale R.Br.) Supplementation in Adults with Low-to-Moderate Cardiovascular Risk: A Randomized Placebo-Controlled Trial
Source: Nutrients. 2026 Aug 5;18(15):2559. doi: 10.3390/nu18152559 (PMC13468134; doi:10.3390/nu18152559)
Supplement: Supplementary file 1 [file nutrients-18-02559-s001.zip › nutrients-4451523-supplementary.pdf]

Supplementary Table S1. Altered pathways following watercress supplementation.

Pathways	Match Status	$-\log_{10}(p)$	Impact
Galactose metabolism	4/27	3.6979	0.0
Fructose and mannose metabolism	1/20	3.1948	0.03313
Glycerolipid metabolism	1/16	2.6645	0.23676
Tryptophan metabolism	10/41	2.5765	0.62645
Purine metabolism	14/70	2.5565	0.30323
Glycolysis or Gluconeogenesis	5/26	2.2697	0.22893
D-Amino acid metabolism	2/15	2.1364	0.0
Tyrosine metabolism	1042	2.0878	0.52405
Sphingolipid metabolism	1/32	2.0333	0.0
Pyruvate metabolism	7/23	1.8204	0.30053
One carbon pool by folate	12/26	1.8135	0.42978
Glycosaminoglycan degradation	1/23	1.6585	0.0
Glycine, serine and threonine metabolism	10/33	1.6539	0.43539
Glutathione metabolism	7/28	1.5805	0.31312
Terpenoid backbone biosynthesis	1/18	1.4805	0.11429
Glyoxylate and dicarboxylate metabolism	10/32	1.459	0.13667
Propanoate metabolism	2/22	1.3552	0.0
Arginine and proline metabolism	8/36	1.2773	0.33023
Pentose phosphate pathway	2/23	1.179	0.19845
Citrate cycle (TCA cycle)	8/20	1.104	0.40618
Vitamin B6 metabolism	1/9	1.0707	0.07843
Pantothenate and CoA biosynthesis	4/20	1.0668	0.0
beta-Alanine metabolism	4/21	1.0635	0.05597
Nicotinate and nicotinamide metabolism	4/15	1.0292	0.37281
Folate biosynthesis	5/27	0.94118	0.21801
Glycerophospholipid metabolism	4/36	0.89139	0.08288
Lipoic acid metabolism	1/28	0.85918	0.0
Amino sugar and nucleotide sugar metabolism	1/42	0.82272	0.0
Alanine, aspartate and glutamate metabolism	12/28	0.76303	0.69792
Histidine metabolism	6/16	0.75528	0.36065
Cysteine and methionine metabolism	8/33	0.74154	0.53894
Valine, leucine and isoleucine biosynthesis	6/8	0.72944	0.0
Arginine biosynthesis	7/14	0.71733	0.50532
Butanoate metabolism	4/15	0.70117	0.11111
Fatty acid biosynthesis	1/47	0.69628	0.0
Valine, leucine and isoleucine degradation	7/40	0.57869	0.03252
Selenocompound metabolism	2/20	0.46892	0.06818
Ubiquinone and other terpenoid-quinone biosynthesis	2/19	0.45999	0.0
Ether lipid metabolism	1/20	0.39367	0.0
Phenylalanine, tyrosine and tryptophan biosynthesis	2/4	0.37962	1.0
Phenylalanine metabolism	2/8	0.37962	0.35714
Pyrimidine metabolism	3/39	0.32927	0.04423
Steroid biosynthesis	1/41	0.31938	0.02837
Steroid hormone biosynthesis	1/87	0.31938	0.00523
Thiamine metabolism	1/7	0.2748	0.0

**Pathways**	**Match Status**	**$-\log_{10}(p)$**	**Impact**
Ascorbate and aldarate metabolism	2/9	0.27198	0.52381
Inositol phosphate metabolism	2/30	0.27198	0.12939
Lysine degradation	3/30	0.19718	0.0
Biotin metabolism	1/10	0.14303	0.0
Primary bile acid biosynthesis	2/46	0.11406	0.05823
Pentose and glucuronate interconversions	3/19	0.10558	0.26506
Taurine and hypotaurine metabolism	3/8	0.091649	0.82857
Porphyrin metabolism	2/31	0.079627	0.02795
Nitrogen metabolism	2/6	0.054854	0.0
